# Construction of a Nomogram Model for Predicting Pathologic Complete Response in Breast Cancer Neoadjuvant Chemotherapy Based on the Pan-Immune Inflammation Value

**DOI:** 10.3390/curroncol32040194

**Published:** 2025-03-27

**Authors:** Zhuowan Tian, Yiqing Xi, Mengting Chen, Meishun Hu, Fangfang Chen, Lei Wei, Jingwei Zhang

**Affiliations:** 1 Hubei Key Laboratory of Tumor Biological Behaviors, Department of Breast and Thyroid Surgery, Hubei Cancer Clinical Study Center, Zhongnan Hospital, Wuhan University, Wuhan 430071, China; tianzw98188@whu.edu.cn (Z.T.); chenmt@whu.edu.cn (M.C.); hms522082343@gmail.com (M.H.); chenfangfang@znhosptial.cn (F.C.); 2 Department of Head and Neck Surgery, Hubei Cancer Hospital, Tongji Medical College, Huazhong University of Science and Technology, Wuhan 430079, China; xiyq17@whu.edu.cn; 3 Hubei Provincial Key Laboratory of Developmentally Originated Disease, Department of Pathology and Pathophysiology, School of Basic Medical Sciences, Wuhan University, Wuhan 430071, China; leiwei@whu.edu.cn

**Keywords:** breast cancer, pan-immune inflammation value (PIV), pathologic complete response (pCR), neoadjuvant chemotherapy (NAC), nomogram

## Abstract

Background: The pan-immune inflammation value (PIV) has unclear predictive utility for pathologic complete response (pCR) in breast cancer patients undergoing neoadjuvant chemotherapy (NAC). This study aimed to evaluate the PIV’s predictive value and develop a nomogram integrating PIV for individualized pCR prediction. Methods: In a retrospective multicenter study of 507 NAC-treated patients (training cohort: 357; validation cohort: 150), independent predictors of pCR were identified through univariate and multivariate logistic regression. A nomogram was constructed and validated using receiver operating characteristic (ROC) curves, calibration curves, and decision curve analysis (DCA). Net reclassification improvement (NRI) and integrated discrimination improvement (IDI) evaluated the improvement in performance after incorporating the PIV indicator. Results: The high PIV patients (cutoff: 316.533) had significantly lower pCR rates than the low PIV patients (*p* < 0.001). The nomogram incorporating PIV, estrogen receptor (ER), human epidermal growth factor receptor-2 (Her2), tumor diameter, clinical node stage, and chemotherapy regimen showed excellent discrimination (training cohort area under the curve (AUC): 0.861, 95% confidence interval (CI): 0.821–0.901; validation cohort AUC: 0.815, 95% CI: 0.748–0.882). The calibration curves demonstrate high prediction accuracy (Hosmer–Lemeshow test: *p* > 0.05), while DCA, NRI (0.341, 95% CI: 0.181–0.500), and IDI (0.017, 95% CI: 0.004–0.029) confirm clinical utility. Conclusions: The PIV is an independent predictor of pCR, and the PIV-based nomogram provides a reliable tool for optimizing NAC response prediction in breast cancer.

## 1. Introduction

Breast cancer is the second most prevalent malignant tumor among women worldwide, second only to lung cancer. According to the latest global cancer statistics, in 2022, there were approximately 2.3 million new cases and 670,000 deaths from female breast cancer worldwide, accounting for 23.8% of all new cancer cases and 15.4% of cancer-related deaths among women [1,2]. The incidence of breast cancer is highest in countries with a high human development index (HDI), while the mortality rate is significantly higher in low HDI countries [3]. This imbalance in burden reflects significant regional disparities. As the burden of breast cancer continues to rise, its impact is more severe in resource-limited regions.

Neoadjuvant chemotherapy (NAC) refers to systemic chemotherapy administered to breast cancer patients without distant metastasis before surgical treatment or localized radiation therapy. Since its first application in locally advanced breast cancer in the 1970s, NAC has gradually become an essential component of breast cancer treatment, particularly with chemotherapy regimens based on anthracyclines and taxanes. The primary goal of NAC is to reduce tumor burden and provide surgical opportunities for patients with locally advanced breast cancer. With the advancement of clinical research, the indications for NAC have gradually expanded. In addition to patients with locally advanced or inoperable breast cancer, early-stage breast cancer patients have also been included in the treatment scope. For these early-stage operable patients, NAC has been proven to be feasible and can significantly improve postoperative therapeutic outcomes. Currently, NAC strategies not only include traditional chemotherapy drugs but also incorporate novel therapies, such as targeted therapy and immunotherapy. For example, Her2-positive breast cancer patients have commonly been treated with anti-Her2 therapies, such as trastuzumab and pertuzumab. In triple-negative breast cancer (TNBC), the combination of immunotherapy and chemotherapy has shown positive results in multiple clinical trials. Furthermore, for hormone receptor (HR)-positive and Her2-positive patients, the combination of endocrine therapy and targeted therapy has become an effective treatment strategy. Overall, NAC for breast cancer not only helps reduce tumor burden, providing more opportunities for breast-conserving surgery [4], but also reduces the need for axillary lymph node dissection (ALND) [5]. Additionally, it offers valuable information about tumor drug sensitivity, which guides subsequent treatment decisions. As treatment strategies continue to evolve, the application of personalized treatment plans has become a growing trend in neoadjuvant therapy for breast cancer, further improving therapeutic outcomes and long-term prognosis for patients. It also helps assess tumor sensitivity to chemotherapeutic drugs. The achievement of pathologic complete response (pCR) after NAC is correlated with better long-term prognosis [6].

However, as a heterogeneous disease [7], breast cancer exhibits significant differences in treatment response. Current NAC strategies based on intrinsic molecular subtypes often fail to fully reflect patients’ treatment status. Disease progression during NAC is not uncommon, making early identification of unsatisfactory chemotherapy responses essential for timely treatment adjustments and optimal surgical timing.

Inflammation plays a vital role in tumor initiation and progression [8]. Immune-inflammatory biomarkers (IIBs) derived from peripheral blood, such as the neutrophil-to-lymphocyte ratio (NLR) and the platelet-to-lymphocyte ratio (PLR), indicate systemic inflammation and correlate with poor prognosis in cancer patients [9,10,11]. The pan-immune inflammation value (PIV), a novel biomarker, integrates multiple blood cell subsets for a comprehensive view of antitumor inflammatory regulation. PIV has proven to be a superior prognostic predictor in metastatic colorectal cancer [12]. As a simple, objective, and cost-effective indicator, the reliable data currently available on its use in predicting pCR in breast cancer patients after NAC are limited.

This study evaluates the PIV’s predictive value for pCR in breast cancer patients receiving NAC and aims to develop a nomogram model incorporating clinicopathological features to predict chemotherapy response. This research could guide clinical decision making and optimize treatment strategies.

## 2. Materials and Methods

### 2.1. Study Population

This retrospective, multi-center study included cancer patients who underwent NAC at Zhongnan Hospital of Wuhan University between January 2017 and August 2024, and at Hubei Cancer Hospital between January 2023 and January 2024. Patients from Zhongnan Hospital were used as the training cohort, while those from Hubei Cancer Hospital served as the external validation cohort.

The inclusion criteria were as follows: (1) female patients with unilateral primary invasive breast cancer; (2) peripheral blood tests completed within two weeks before the first chemotherapy, with complete clinical records; (3) patients who meet the clinical indications for NAC and have completed at least three cycles of chemotherapy; (4) no history of other primary malignancies.

The exclusion criteria were as follows: (1) pregnancy or lactation; (2) stage IV breast cancer with distant metastasis before NAC; (3) systemic inflammatory diseases, hematologic or autoimmune diseases, or long-term use of corticosteroids or immunosuppressants; (4) preoperative immunotherapy or endocrine therapy; (5) frailty with severe organic diseases involving the heart, lungs, or brain.

This study was approved by the Ethics Committee of Zhongnan Hospital of Wuhan University (Ethics No. 2024278K).

### 2.2. Collection and Definition of Research Indicators

The basic information of the enrolled patients was retrieved from the hospital information system, encompassing the age at diagnosis and menstrual status. Peripheral blood indicators within two weeks prior to their first chemotherapy were recorded, including the absolute counts of lymphocytes, neutrophils, monocytes, and platelets, presented as cells/L. Tumor marker indices prior to chemotherapy, including carbohydrate antigen 15-3 (CA153) and carcinoembryonic antigen (CEA), were documented. The imaging examination at the time of initial diagnosis was reviewed according to the eighth edition of the American Joint Committee on Cancer (AJCC) criteria [13] to evaluate the primary lesions and the overall condition of the patients, recording the lesion number, location, tumor diameter, and clinical nodal stage of the lesions. Additionally, according to the inclusion and exclusion criteria, patients with clinical or radiographic evidence of distant metastases at diagnosis (i.e., stage IV: any T, any N, M1) were excluded, as these patients do not meet the indications for NAC. The pathological results of puncture and surgical specimens were recorded, including the histological grade, pathological type, androgen receptor (AR), estrogen receptor (ER), progesterone receptor (PR), human epidermal growth factor receptor-2 (Her2), and Ki67. A positive result for AR, ER, and PR is defined as positive staining in ≥1% of invasive cancer cells detected by immunohistochemistry (IHC) [14]. Ki67 status was determined using a 20% cutoff value, while a positive Her2 result is defined as 3+ by IHC or 2+ by IHC, with a positive result from in situ hybridization (ISH) [15]. The treatment modalities of the patients were also recorded, including chemotherapy regimens, cycles, as well as breast and axillary surgical procedures. In the selection of chemotherapy regimens, for Her2-positive breast cancer, the treatment regimens included anthracycline + cyclophosphamide followed by taxanes + targeted therapy, or taxanes + carboplatin + targeted therapy, or taxanes + targeted therapy. For triple-negative and HR-positive breast cancers, the regimens included anthracycline + cyclophosphamide followed by taxanes, or anthracycline + cyclophosphamide + taxanes, or anthracycline + cyclophosphamide, or taxanes + cyclophosphamide. Since the chemotherapy drugs mainly used in these regimens are anthracyclines and taxanes, for the convenience of analysis, this study categorized the chemotherapy regimens into three groups: anthracycline-based, taxane-based, and the combination of both. All Her2-positive patients included in this study received trastuzumab and pertuzumab targeted therapy in addition to chemotherapy, in accordance with standard clinical practice guidelines. However, due to economic constraints or concerns about the treatment intensity, some patients could only receive single-agent Her2-targeted therapy. Nonetheless, all Her2-positive patients received at least one form of Her2-targeted therapy. After completing the chemotherapy cycles, depending on the clinical response of primary breast cancer, breast-conserving surgery or total mastectomy was planned as appropriate. Axillary management methods included sentinel lymph node biopsy (SLNB) and ALND. For patients undergoing SLNB, if intraoperative frozen pathological sections confirmed the residual tumor burden of macrometastasis, micrometastasis, or isolated tumor cells (ITC) in the sentinel lymph nodes, complete ALND was required [16].

### 2.3. Determination of the Optimal PIV Cutoff Value

The PIV value was calculated as follows: (neutrophils × monocytes × platelets)/lymphocytes. Given the absence of validated PIV cutoff values in the previous literature, the receiver operating characteristic (ROC) curve was used to calculate the Youden index (Sensitivity + Specificity − 1) based on the training cohort data (Figure S1). The PIV value corresponding to the maximum Youden index (316.533) was selected as the optimal cutoff value, with sensitivity at 88.9% and specificity at 28.1%. The continuous variable was subsequently transformed into a binary variable, categorized into the low PIV group (PIV ≤ 316.533) and the high PIV group (PIV > 316.533) for further analysis.

### 2.4. Evaluation of Efficacy of Neoadjuvant Chemotherapy

During the neoadjuvant chemotherapy period, all enrolled patients underwent clinical physical examination assessments at each treatment cycle and imaging evaluations every two cycles to assess the primary breast lesions and regional lymph nodes, thereby monitoring treatment response. According to the Response Evaluation Criteria in Solid Tumors (RECIST) 1.1 [17], treatment responses were categorized as complete response, partial response, progressive disease, and stable disease. For patients assessed as progressive disease during chemotherapy, surgical intervention should be promptly considered. If the assessment indicates stable disease, the decision to either switch chemotherapy regimens or proceed with surgery should be based on the patient’s specific condition. For patients assessed as partial response or complete response, completion of the planned neoadjuvant chemotherapy course is recommended, even if significant tumor regression is observed, unless the patient is intolerant or strongly requests early surgical intervention. The final evaluation of pathological complete response status after neoadjuvant chemotherapy should be conducted through a comprehensive review of the pathological reports of all enrolled patients following definitive surgery. Pathologic complete response after NAC was defined as the absence of residual invasive carcinoma in both the completely resected breast specimens and all sampled regional lymph nodes (ypT0/Tis and ypN0) [18]. Residual ductal carcinoma in situ within the primary breast lesion was permitted. However, residual ITC in the lymph nodes did not count as pCR. In the patients’ pathological reports, pCR was presented as Miller–Payne (MP) grade 5, along with ypN0, or a residual cancer burden (RCB) score of 0 [19].

### 2.5. Statistical Analysis

Statistical analyses were conducted using IBM SPSS (version 27.0) and RStudio (version 4.3.3). Categorical variables were presented as frequencies and percentages. Group comparisons were performed via the Chi-square test or Fisher’s exact test. Univariate logistic regression was used to identify potential predictors. Significant variables underwent multicollinearity analysis. Variables without severe multicollinearity were included in multivariate logistic regression for further selection. Subsequently, the nomogram prediction model was constructed using the “rms” and “regplot” packages. To comprehensively evaluate the predictive performance of the model, the “pROC” package was used to plot the ROC curves, measuring the discriminative ability of the model. The “rms” package was used to draw the calibration curve, evaluating the consistency between the predicted probabilities and the actual observed values. The Hosmer–Lemeshow test, implemented via the “ResourceSelection” package, evaluated the goodness-of-fit of the model. The “rmda” package generated the decision curve analysis (DCA) curve, enabling a comprehensive consideration of the model’s clinical utility. Furthermore, with the “nricens” package, the net reclassification improvement (NRI) and integrated discrimination improvement (IDI) were calculated to evaluate the enhancement of the model’s performance after incorporating the PIV indicator. Statistical significance was determined by two-sided tests, with *p* < 0.05 considered statistically significant. The levels of significance are defined as follows: * *p* < 0.05, ** *p* < 0.01, and *** *p* < 0.001.

## 3. Results

### 3.1. Baseline Characteristics of Enrolled Patients

**Figure 1:**
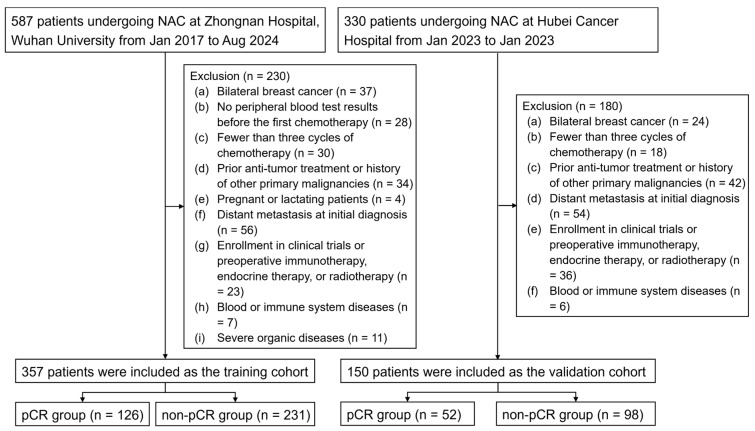
The patient selection process for this study. Abbreviations: pCR: pathologic complete response; NAC: neoadjuvant chemotherapy.

A total of 507 patients were included in this study based on the inclusion and exclusion criteria, with 357 cases from Zhongnan Hospital of Wuhan University assigned to the training cohort and 150 cases from Hubei Cancer Hospital assigned to the external validation cohort (Figure 1). Table 1 provides detailed information on the clinical characteristics, pathological features, treatment approaches, and chemotherapy responses of the two cohorts. In the training cohort, 35.29% (126/357) of the patients achieved pCR after chemotherapy, compared to 34.67% (52/150) in the validation cohort. No significant differences were observed between the training and validation cohorts in any of these characteristics (all *p* > 0.05), ensuring a reliable foundation for subsequent analyses. Considering that Her2-positive breast cancer patients require trastuzumab and pertuzumab targeted therapy in addition to chemotherapy, we compared the baseline characteristics of Her2-positive patients receiving targeted therapy in the training and validation cohorts (Table S1). The results show that 82.91% (131/158) of the patients in the training cohort received dual-agent targeted therapy, while 87.18% (68/78) of the patients in the validation cohort received dual-agent therapy. No significant difference was observed between the two cohorts (*p* > 0.05).

### 3.2. Association Analysis of PIV with Clinicopathological Characteristics and Chemotherapy Response

Analysis of the 357 samples in the training cohort revealed that the PIV level was significantly correlated with CA153, age, menstrual status, pathological type, Her2 status, tumor diameter, pCR status, and pathological nodal status after NAC (*p* < 0.05). Specifically, in the low PIV group, 60.43% (168/278) of the patients achieved axillary lymph node negative status, and 40.29% (112/278) achieved pCR. In contrast, in the high PIV group, only 44.30% (35/79) of the patients achieved axillary lymph node negative status, and 17.72% (14/79) achieved pCR (Table 2).

### 3.3. Identification of Independent Influencing Factors for pCR After NAC

The univariate logistic regression analysis indicated the CA153, PIV, grade, pathological type, chemotherapy cycles, ER status, PR status, Her2 status, Ki67 level, tumor diameter, clinical nodal stage, and chemotherapy regimen as significant factors associated with achieving pCR (all *p* < 0.05).

The correlation matrix confirmed the absence of multicollinearity among these variables (Figure S2). Consequently, all significant factors were incorporated into the multivariate logistic regression analysis. The results reveal that the high PIV group (odds ratio (OR): 0.349, 95% confidence interval (CI): 0.149–0.778, *p* = 0.012), ER-positive (OR: 0.467, 95% CI: 0.220–0.972, *p* = 0.044), Her2-positive (OR: 4.529, 95% CI: 2.252–9.304, *p* < 0.001), tumor diameter > 5 cm (OR: 0.238, 95% CI: 0.077–0.715, *p* = 0.011), initial axillary lymph node stage 3 (OR: 0.269, 95% CI: 0.105–0.665, *p* = 0.005), and taxane-based chemotherapy (OR: 3.841, 95% CI: 1.848–8.215, *p* < 0.001) were identified as independent factors influencing the achievement of pCR after NAC (Table 3).

### 3.4. Nomogram Prediction Model Development and Evaluation

**Figure 2:**
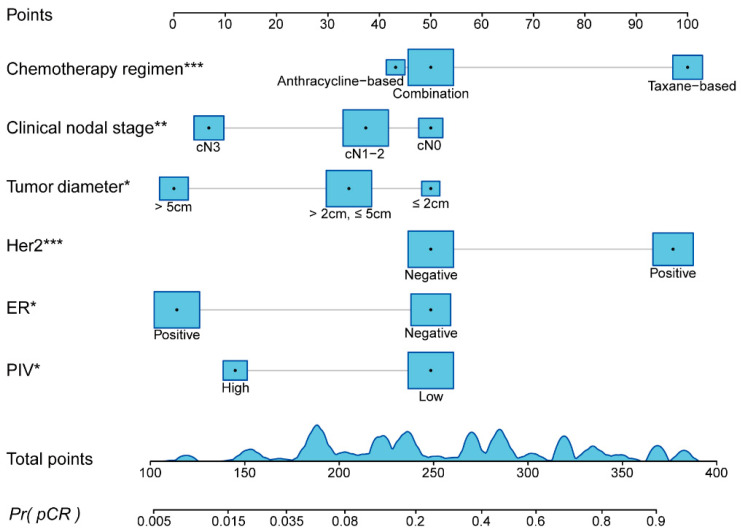
Nomogram for predicting the probability of achieving pathologic complete response after neoadjuvant chemotherapy. Abbreviations: PIV: pan-immune inflammation value; ER: estrogen receptor; Her2: human epidermal growth factor receptor-2; cN: clinical nodal stage. The levels of significance are defined as follows: * *p* < 0.05, ** *p* < 0.01, and *** *p* < 0.001.

A nomogram was constructed based on the six key variables identified through screening to visually illustrate their roles in predicting pCR after NAC for breast cancer (Figure 2). These six key variables were selected based on their predictive value and assigned corresponding point values. Specifically, the chemotherapy regimen was categorized into anthracycline-based (43 points), combination (50 points), and taxane-based (100 points). The clinical nodal stage was divided into cN0 (50 points), cN1-2 (37 points), and cN3 (7 points). The tumor diameter was classified into three groups: ≤2 cm (50 points), >2 cm and ≤5 cm (34 points), and >5 cm (0 points). Her2 status was assigned 50 points for negative and 97 points for positive, while ER status was assigned 50 points for negative and 1 point for positive. The PIV variable was categorized into low (50 points) and high (12 points). In the nomogram, variable names are listed on the left, while variable values are annotated on the right. The points of each variable can be determined according to its corresponding value. The total points, derived by summing the individual points, corresponds to the predicted probability of achieving pCR.

**Figure 3:**
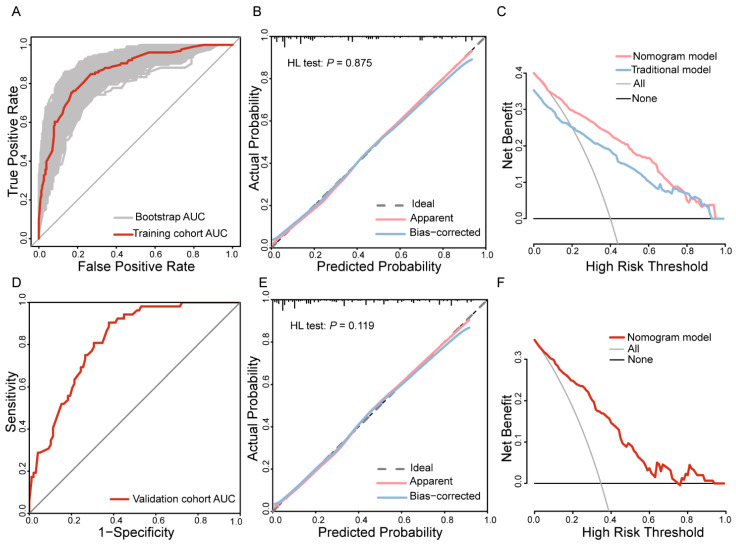
Development and validation of the nomogram predictive model for pathologic complete response after neoadjuvant chemotherapy. For the training cohort, the receiver operating characteristic (ROC) curve and bootstrap ROC curve (**A**), calibration curve and Hosmer–Lemeshow test results (**B**), and decision curve analysis (DCA) comparing the combined model and the traditional model (**C**). For the validation cohort, the ROC curve (**D**), calibration curve (**E**), and DCA (**F**). Abbreviations: PIV: pan-immune inflammation value; AUC: area under the curve; CI: confidence interval.

In the training cohort, the area under the curve (AUC) of the nomogram for predicting pCR was 0.861 (95%CI: 0.821–0.901), with a specificity of 83.1% and a sensitivity of 75.4%. Internal validation using the bootstrap method yielded a mean AUC of 0.861 (95%CI: 0.822–0.900) (Figure 3A). Moreover, the calibration curve showed that the actual curve was close to the ideal curve, and the predicted probability of the model was highly consistent with the actual probability (Hosmer–Lemeshow test: *p* = 0.875) (Figure 3B). These results confirm that the model has good discrimination and calibration.

To evaluate the improvement in model performance by incorporating the PIV indicator, this study compared a six-indicator combined predictive model that includes PIV with a traditional model based only on clinicopathological variables (ER, Her2, tumor diameter, clinical nodal stage, and chemotherapy regimen). The traditional model was constructed by excluding the peripheral blood inflammation marker PIV while using the remaining significant clinicopathological indicators to develop the nomogram and calculate the predicted risk score for each patient. The results show that the combined model achieved a continuous NRI of 0.341 (95% CI: 0.181–0.500) and an IDI of 0.017 (95% CI: 0.004–0.029), both significantly better than the traditional model (*p* < 0.001).

Moreover, DCA demonstrated that the combined model provided higher net benefits across a broader range of risk thresholds (Figure 3C), indicating that the nomogram incorporating the PIV indicator could more accurately predict the likelihood of achieving pCR after NAC and has significant clinical utility. However, although the AUC of the combined model (0.861 vs. 0.856 for the traditional model) was slightly higher, DeLong’s test revealed no statistically significant difference between the two models (*p* = 0.355) (Figure S3).

### 3.5. Validation of the Nomogram

This study further validated the model using an independent dataset from an external center. The model demonstrated strong discriminative ability, achieving an AUC of 0.815 (95%CI: 0.748–0.882) (Figure 3D). The calibration curve indicates high consistency between the predicted and observed probabilities (Hosmer–Lemeshow test: *p* = 0.119) (Figure 3E), reflecting excellent calibration performance. In addition, DCA showed a relatively high net benefit across a wide range of risk thresholds (Figure 3F). In summary, the model exhibited robust performance in the training cohort and demonstrated good reliability and generalizability in the external independent dataset.

## 4. Discussion

The tumor microenvironment (TME), as a complex and dynamic ecosystem, is composed of various components, including immune cells, cancer-associated fibroblasts (CAFs), endothelial cells (ECs), and the extracellular matrix (ECM). Changes in these components play a crucial role in cancer progression [20]. Whether as a result of prolonged chronic inflammation or the early stages of tumor initiation, tumors at different stages of progression either repel or induce various cells, including T cells, natural killer (NK) cells, and dendritic cells (DCs). In addition, the influence of tumor-stimulated myeloid cells, particularly the recruitment and activation of macrophages and neutrophils, supports the inflammatory environment that facilitates tumor progression [21,22,23,24,25]. Therefore, any pre-treatment systemic inflammatory state can serve as crucial information for assessing cancer treatment response and prognostic progression.

Peripheral blood cell-derived IIBs, such as the NLR, PLR, systemic inflammation response index (SIRI) [26], and systemic immune inflammation index (SII) [27], can reflect the current inflammatory and immune status of patients, providing valuable information for prognostic prediction in various solid tumors. For breast cancer specifically, multiple studies have discussed the role of NLR in predicting tumor prognosis [28,29]. However, the relationship between tumors and the host, as well as between inflammation and immunity, is complex. The peripheral blood ratios derived from simple calculations have limitations in discriminative ability, which restricts their clinical application. A meta-analysis including 45 studies showed that in breast cancer patients receiving neoadjuvant chemotherapy, there was no significant association between survival rate and NLR [30]. Therefore, finding a more comprehensive indicator that reflects the overall systemic inflammation and immune system activation status of patients is of great significance.

The peripheral blood-derived PIV indicator encompasses neutrophils, monocytes, platelets, and lymphocytes, potentially providing a more comprehensive reflection of the patient’s systemic inflammation and immune system activation. It serves as a new marker for evaluating treatment response and prognosis. The PIV amplifies the effect of individual cell counts, accurately capturing subtle changes in specific immune cell populations related to clinical outcomes, and its prognostic ability surpasses that of other common IIB [12]. The pre-treatment PIV has been shown to be associated with pCR in tumors such as esophageal squamous cell carcinoma [31] and non-small-cell lung cancer [32]. Gasparri [33] found that low PIV levels could predict axillary pCR in breast cancer patients receiving NAC. Additionally, the prognostic value of PIV in tumor patient survival has also been confirmed, with meta-analyses indicating that high PIV levels are associated with shorter overall survival (OS) and disease-free survival (DFS) in patients with digestive system tumors [34]. Other studies have also highlighted its relevance in the prognosis of nasopharyngeal carcinoma [35]. Moreover, this study found that the PIV indicator is closely related to the patient’s age and menstrual status. Younger, premenopausal breast cancer patients typically have higher PIV values. Shah’s [36] research indicates that, compared to older patients, younger patients’ tumors are more likely to exhibit mutations associated with aggressive disease and have a higher number of immune infiltrating cells, suggesting a stronger inflammatory and immune response to the tumor in younger patients. Therefore, this study offers a new perspective on using PIV to assess early inflammation and immune status in patients.

This study conducted a retrospective analysis using real-world data to evaluate and validate the predictive value of the PIV for the efficacy of NAC in breast cancer patients. The results confirm that low pre-chemotherapy PIV levels are associated with a higher pCR, providing new clinical evidence for the use of the PIV in predicting chemotherapy response in patients.

Currently, the decision making for neoadjuvant therapy in breast cancer is mainly based on the clinical and pathological characteristics of the tumor, without fully considering the host’s systemic immune-inflammatory status. Even for the same tumor subtype, treatment response and prognosis can vary among different patients. This study developed a combined model that includes six indicators: PIV, ER, Her2, tumor diameter, clinical nodal stage, and chemotherapy regimen, to predict the pCR in patients receiving NAC. The model demonstrates good predictive accuracy, excellent calibration ability, and significant clinical applicability, providing a reliable and efficient tool for predicting pCR after NAC. Moreover, the model’s predictive capability was enhanced compared to traditional prediction models by incorporating the PIV. Additionally, this study validated the nomogram using an external center dataset, confirming that the model has high reliability and generalizability, which can serve as a reference for clinical decision making. In future research, we will further validate the clinical application value of this model.

This study has several limitations. There is a lack of consensus among different research institutions regarding the cutoff values for the PIV indicator. Although this study validated the model using data from an external institution, a reliable and universally applicable cutoff value definition is still needed to more accurately stratify peripheral blood IIBs. Furthermore, due to the retrospective design of this study, there is an inevitable risk of selection bias. Further prospective studies are needed to validate the effectiveness of this indicator in predicting pCR.

## 5. Conclusions

In conclusion, this study confirms that pre-chemotherapy PIV levels were an independent predictor of pCR in breast cancer patients after NAC. Based on six indicators with independent predictive values, namely PIV, ER, Her2, tumor diameter, clinical nodal stage, and chemotherapy regimen, a nomogram model was constructed for predicting post-chemotherapy pCR. The model performed well in the training cohort, with an AUC value of 0.861 (95%CI: 0.821–0.901), and the calibration curve showed a high degree of agreement between the predictions and observations (Hosmer–Lemeshow test: *p* = 0.875). DCA, NRI, and IDI further verified the clinical application value of this model and its superiority over traditional models. In addition, the model also shows good stability and accuracy when verified by the data of external institutions. This study provides a theoretical basis for evaluating the inflammatory and immune status of breast cancer patients, and helps to identify patients who are not sensitive to chemotherapy early, thereby supporting timely adjustment of treatment regimen and determining the best time to operate. In addition, this study provides a new tool for evaluating the efficacy of NAC, which helps to optimize the strategy of NAC for breast cancer and improve the overall treatment effect.

## Figures and Tables

**Table 1:** Comparison of baseline characteristics between the training and validation cohorts.

Characteristics	Total (n = 507), n (%)	Training Cohort (n = 357), n (%)	Validation Cohort (n = 150), n (%)	*p* Value
CA153 (U/mL)				0.324
≤28	409 (80.67)	292 (81.79)	117 (78.00)	
>28	98 (19.33)	65 (18.21)	33 (22.00)	
CEA (ng/mL)				0.805
≤5	433 (85.40)	304 (85.15)	129 (86.00)	
>5	74 (14.60)	53 (14.85)	21 (14.00)	
PIV				0.346
Low	389 (76.73)	278 (77.87)	111 (74.00)	
High	118 (23.27)	79 (22.13)	39 (26.00)	
Age (years)				0.947
≤50	276 (54.44)	194 (54.34)	82 (54.67)	
>50	231 (45.56)	163 (45.66)	68 (45.33)	
Menstrual status				0.666
Pre-menopausal	261 (51.48)	186 (52.10)	75 (50.00)	
Post-menopausal	246 (48.52)	171 (47.90)	75 (50.00)	
Lesion number				0.676
Single lesion	355 (70.02)	248 (69.47)	107 (71.33)	
Multiple lesions	152 (29.98)	109 (30.53)	43 (28.67)	
Grade				0.213
Grades I–II	324 (63.91)	222 (62.18)	102 (68.00)	
Grade III	183 (36.09)	135 (37.82)	48 (32.00)	
Pathological type				0.518
IDC	437 (86.19)	310 (86.83)	127 (84.67)	
Others	70 (13.81)	47 (13.17)	23 (15.33)	
Chemotherapy cycles				0.134
≤4	73 (14.40)	46 (12.89)	27 (18.00)	
>4	434 (85.60)	311 (87.11)	123 (82.00)	
AR				0.897
Negative	93 (18.34)	66 (18.49)	27 (18.00)	
Positive	414 (81.66)	291 (81.51)	123 (82.00)	
ER				0.593
Negative	224 (44.18)	155 (43.42)	69 (46.00)	
Positive	283 (55.82)	202 (56.58)	81 (54.00)	
PR				0.323
Negative	274 (54.04)	198 (55.46)	76 (50.67)	
Positive	233 (45.96)	159 (44.54)	74 (49.33)	
Her2				0.111
Negative	271 (53.45)	199 (55.74)	72 (48.00)	
Positive	236 (46.55)	158 (44.26)	78 (52.00)	
Ki67				0.598
≤20%	91 (17.95)	62 (17.37)	29 (19.33)	
>20%	416 (82.05)	295 (82.63)	121 (80.67)	
Location				0.359
Central region	66 (13.02)	49 (13.73)	17 (11.33)	
Upper outer quadrant	235 (46.35)	169 (47.34)	66 (44.00)	
Upper inner quadrant	90 (17.75)	56 (15.69)	34 (22.67)	
Lower inner quadrant	39 (7.69)	26 (7.28)	13 (8.67)	
Lower outer quadrant	77 (15.19)	57 (15.97)	20 (13.33)	
Tumor diameter				0.846
≤2 cm	51 (10.06)	37 (10.36)	14 (9.33)	
>2 cm, ≤5 cm	322 (63.51)	228 (63.87)	94 (62.67)	
>5 cm	134 (26.43)	92 (25.77)	42 (28.00)	
Clinical nodal stage				0.202
cN0	88 (17.36)	59 (16.53)	29 (19.33)	
cN1-2	302 (59.57)	208 (58.26)	94 (62.67)	
cN3	117 (23.08)	90 (25.21)	27 (18.00)	
Chemotherapy regimen				0.157
Anthracycline–taxane combination	307 (60.55)	223 (62.46)	84 (56.00)	
Taxane-based	149 (29.39)	96 (26.89)	53 (35.33)	
Anthracycline-based	51 (10.06)	38 (10.64)	13 (8.67)	
pCR status				0.893
pCR	178 (35.11)	126 (35.29)	52 (34.67)	
Non-pCR	329 (64.89)	231 (64.71)	98 (65.33)	
ypN status				0.553
ypN-negative	284 (56.02)	203 (56.86)	81 (54.00)	
ypN-positive	223 (43.98)	154 (43.14)	69 (46.00)	
Breast surgery				0.919
Breast-conserving	53 (10.45)	37 (10.36)	16 (10.67)	
Mastectomy	454 (89.55)	320 (89.64)	134 (89.33)	
Axillary surgery				0.268
SLNB	56 (11.05)	43 (12.04)	13 (8.67)	
ALND	451 (88.95)	314 (87.96)	137 (91.33)	

Abbreviations: CA153: carbohydrate antigen 15-3; CEA: carcinoembryonic antigen; PIV: pan-immune inflammation value; IDC: invasive ductal carcinoma; AR: androgen receptor; ER: estrogen receptor; PR: progesterone receptor; Her2: human epidermal growth factor receptor-2; cN: clinical nodal stage; pCR: pathologic complete response; ypN: post-neoadjuvant pathological nodal staging; SLNB: sentinel lymph node biopsy; ALND: axillary lymph node dissection.

**Table 2:** Association analysis of pan-immune inflammation value (PIV) with clinicopathological features and chemotherapy response.

Characteristics	Training Cohort (n = 357), n (%)	Low PIV (n = 278), n (%)	High PIV (n = 79), n (%)	*p*-Value
CA153 (U/mL)				<0.001 ***
≤28	292 (81.79)	238 (85.61)	54 (68.35)	
>28	65 (18.21)	40 (14.39)	25 (31.65)	
CEA (ng/mL)				0.794
≤5	304 (85.15)	236 (84.89)	68 (86.08)	
>5	53 (14.85)	42 (15.11)	11 (13.92)	
Age (years)				0.039 *
≤50	194 (54.34)	143 (51.44)	51 (64.56)	
>50	163 (45.66)	135 (48.56)	28 (35.44)	
Menstrual status				0.006 **
Pre-menopausal	186 (52.10)	134 (48.20)	52 (65.82)	
Post-menopausal	171 (47.90)	144 (51.80)	27 (34.18)	
Lesion number				0.090
Single lesion	248 (69.47)	187 (67.27)	61 (77.22)	
Multiple lesions	109 (30.53)	91 (32.73)	18 (22.78)	
Grade				0.767
Grades I–II	222 (62.18)	174 (62.59)	48 (60.76)	
Grade III	135 (37.82)	104 (37.41)	31 (39.24)	
Pathological type				0.035 *
IDC	310 (86.83)	247 (88.85)	63 (79.75)	
Others	47 (13.17)	31 (11.15)	16 (20.25)	
AR				0.265
Negative	66 (18.49)	48 (17.27)	18 (22.78)	
Positive	291 (81.51)	230 (82.73)	61 (77.22)	
ER				0.173
Negative	155 (43.42)	126 (45.32)	29 (36.71)	
Positive	202 (56.58)	152 (54.68)	50 (63.29)	
PR				0.217
Negative	198 (55.46)	159 (57.19)	39 (49.37)	
Positive	159 (44.54)	119 (42.81)	40 (50.63)	
Her2				0.005 **
Negative	199 (55.74)	144 (51.80)	55 (69.62)	
Positive	158 (44.26)	134 (48.20)	24 (30.38)	
Ki67				0.443
≤20%	62 (17.37)	46 (16.55)	16 (20.25)	
>20%	295 (82.63)	232 (83.45)	63 (79.75)	
Location				0.919
Central region	49 (13.73)	36 (12.95)	13 (16.46)	
Upper outer quadrant	169 (47.34)	133 (47.84)	36 (45.57)	
Upper inner quadrant	56 (15.69)	43 (15.47)	13 (16.46)	
Lower inner quadrant	26 (7.28)	20 (7.19)	6 (7.59)	
Lower outer quadrant	57 (15.97)	46 (16.55)	11 (13.92)	
Tumor diameter				0.038 *
≤2 cm	37 (10.36)	31 (11.15)	6 (7.59)	
>2 cm, ≤5 cm	228 (63.87)	184 (66.19)	44 (55.70)	
>5 cm	92 (25.77)	63 (22.66)	29 (36.71)	
Clinical nodal stage				0.260
cN0	59 (16.53)	50 (17.99)	9 (11.39)	
cN1-2	208 (58.26)	162 (58.27)	46 (58.23)	
cN3	90 (25.21)	66 (23.74)	24 (30.38)	
pCR status				<0.001 ***
pCR	126 (35.29)	112 (40.29)	14 (17.72)	
Non-pCR	231 (64.71)	166 (59.71)	65 (82.28)	
ypN status				0.011 *
ypN-negative	203 (56.86)	168 (60.43)	35 (44.30)	
ypN-positive	154 (43.14)	110 (39.57)	44 (55.70)	

Abbreviations: CA153: carbohydrate antigen 15-3; CEA: carcinoembryonic antigen; PIV: pan-immune inflammation value; IDC: invasive ductal carcinoma; AR: androgen receptor; ER: estrogen receptor; PR: progesterone receptor; Her2: human epidermal growth factor receptor-2; cN: clinical nodal stage; pCR: pathologic complete response; ypN: post-neoadjuvant pathological nodal staging. The levels of significance are defined as follows: * *p* < 0.05, ** *p* < 0.01, and *** *p* < 0.001.

**Table 3:** Univariate and multivariate logistic regression analysis in the training cohort to identify factors associated with pathologic complete response.

Characteristics	Univariable		Multivariable	
	Odds Ratio (95%CI)	*p* Value	Odds Ratio (95%CI)	*p* Value
CA153 (U/mL)				
>28 vs. ≤28	0.441 (0.226–0.814)	0.012 *	0.849 (0.366–1.918)	0.696
CEA (ng/mL)				
>5 vs. ≤5	0.549 (0.272–1.045)	0.079		
PIV				
High vs. low	0.319 (0.165–0.581)	<0.001 ***	0.349 (0.149–0.778)	0.012 *
Age (years)				
>50 vs. ≤50	0.839 (0.541–1.298)	0.433		
Menstrual status				
Post-menopausal vs. pre-menopausal	1.032 (0.668–1.594)	0.886		
Lesion number				
Multiple lesions vs. single lesion	1.533 (0.962–2.438)	0.071		
Grade				
Grade III vs. grades I-II	1.891 (1.212–2.956)	0.005 **	1.835 (0.990–3.444)	0.055
Pathological type				
Others vs. IDC	0.452 (0.206–0.910)	0.034 *	0.712 (0.260–1.840)	0.494
Chemotherapy cycles				
>4 vs. ≤4	2.904 (1.375–6.898)	0.009 **	2.255 (0.703–7.604)	0.177
AR				
Positive vs. negative	0.944 (0.545–1.666)	0.840		
ER				
Positive vs. negative	0.210 (0.131–0.332)	<0.001 ***	0.467 (0.220–0.972)	0.044 *
PR				
Positive vs. negative	0.247 (0.150–0.398)	<0.001 ***	0.473 (0.214–1.041)	0.062
Her2				
Positive vs. negative	7.665 (4.724–12.710)	<0.001 ***	4.529 (2.252–9.304)	<0.001 ***
Ki67				
>20% vs. ≤20%	1.890 (1.031–3.643)	0.047 *	1.585 (0.680–3.822)	0.293
Location				
Upper outer quadrant vs. central region	1.646 (0.828–3.437)	0.167		
Upper inner quadrant vs. central region	1.312 (0.565–3.104)	0.530		
Lower inner quadrant vs. central region	1.731 (0.622–4.792)	0.289		
Lower outer quadrant vs. central region	1.741 (0.767–4.062)	0.190		
Tumor diameter				
>2 cm, ≤5 cm vs. ≤2 cm	0.618 (0.306–1.244)	0.176	0.639 (0.249–1.627)	0.347
>5 cm vs. ≤2 cm	0.215 (0.092–0.489)	<0.001 ***	0.238 (0.077–0.715)	0.011 *
Clinical nodal stage				
cN1-2 vs. cN0	0.624 (0.348–1.123)	0.114	0.577 (0.264–1.246)	0.163
cN3 vs. cN0	0.380 (0.188–0.759)	0.007 **	0.269 (0.105–0.665)	0.005 **
Chemotherapy regimen				
Taxane-based vs. anthracycline–taxane combination	9.080 (5.313–15.939)	<0.001 ***	3.841 (1.848–8.215)	<0.001 ***
Anthracycline-based vs. anthracycline–taxane combination	0.511 (0.168–1.272)	0.184	1.394 (0.367–4.739)	0.605

Abbreviations: CA153: carbohydrate antigen 15-3; CEA: carcinoembryonic antigen; PIV: pan-immune inflammation value; IDC: invasive ductal carcinoma; AR: androgen receptor; ER: estrogen receptor; PR: progesterone receptor; Her2: human epidermal growth factor receptor-2; cN: clinical nodal stage. The levels of significance are defined as follows: * *p* < 0.05, ** *p* < 0.01, and *** *p* < 0.001.

## Data Availability

All data generated during this study are included in the article and Supplementary Materials. Further enquiries can be directed to the corresponding author.

## Supplementary Materials

The following supporting information can be downloaded at https://www.mdpi.com/article/10.3390/curroncol32040194/s1: Table S1: Comparison of baseline characteristics of Her2-positive breast cancer patients receiving targeted therapy in the training and validation cohorts. Figure S1: Identification of the best cutoff point of the pan-immune inflammation value (PIV). Figure S2: Constructing a correlation matrix of significant variables from univariate logistic regression to assess multicollinearity. Figure S3: Receiver operating characteristic (ROC) curves of the combined model and the traditional model.

## Abbreviations

The following abbreviations are used in this manuscript:

AJCC	American Joint Committee on Cancer
ALND	Axillary lymph node dissection
AR	Androgen receptor
AUC	Area under the curve
CA153	Carbohydrate antigen 15-3
CAFs	Cancer-associated fibroblasts
CEA	Carcinoembryonic antigen
CI	Confidence interval
cN	Clinical nodal stage
DCA	Decision curve analysis
DCs	Dendritic cells
DFS	Disease-free survival
ECM	Extracellular matrix
ECs	Endothelial cells
ER	Estrogen receptor
HDI	Human development index
Her2	Human epidermal growth factor receptor-2
HR	Hormone receptor
IDC	Invasive ductal carcinoma
IDI	Integrated discrimination improvement
IHC	Immunohistochemistry
IIBs	Immune-inflammatory biomarkers
ISH	In situ hybridization
ITC	Isolated tumor cells
MP	Miller–Payne
NAC	Neoadjuvant chemotherapy
NK cells	Natural killer cells
NLR	Neutrophil-to-lymphocyte ratio
NRI	Net reclassification improvement
OR	Odds ratio
OS	Overall survival
pCR	Pathologic complete response
PIV	Pan-immune inflammation value
PLR	Platelet-to-lymphocyte ratio
PR	Progesterone receptor
RCB	Residual cancer burden
RECIST	Response Evaluation Criteria in Solid Tumors
ROC	Receiver operating characteristic
SII	Systemic immune inflammation index
SIRI	Systemic inflammation response index
SLNB	Sentinel lymph node biopsy
TME	Tumor microenvironment
TNBC	Triple-negative breast cancer
ypN	Post-neoadjuvant pathological nodal staging
