# Supplementary material for: Construction of a Nomogram Model for Predicting Pathologic Complete Response in Breast Cancer Neoadjuvant Chemotherapy Based on the Pan-Immune Inflammation Value
Source: Curr Oncol. 2025 Mar 27;32(4):194. doi: 10.3390/curroncol32040194 (PMC12026318; doi:10.3390/curroncol32040194)
Supplement: Supplementary file 1 [file curroncol-32-00194-s001.zip › curroncol-3476492-supplementary.pdf]

Supplementary Information for

Construction of a nomogram model for predicting pathologic complete response in breast cancer neoadjuvant chemotherapy based on the Pan-immune inflammation value

Zhuowan Tian[1], Yiqing Xi[2], Mengting Chen[1], Meishun Hu[1], Fangfang Chen[1], Lei Wei[3] and Jingwei Zhang[1*]

[1]Department of Breast and Thyroid Surgery, Zhongnan Hospital, Hubei Key Laboratory of Tumor Biological Behaviors, Hubei Cancer Clinical Study Center, Wuhan University, Wuhan, Hubei 430071, China.

[2]Department of Head and Neck Surgery, Hubei Cancer Hospital, Tongji Medical College, Huazhong University of Science and Technology, Wuhan, Hubei 430079, China.

[3]Department of Pathology and Pathophysiology, Hubei Provincial Key Laboratory of Developmentally Originated Disease, School of Basic Medical Sciences, Wuhan University, Wuhan, Hubei 430071, China.

[*]Correspondence: Jingwei Zhang, zjwzhang68@whu.edu.cn

Table S1. Comparison of baseline characteristics of Her2-positive breast cancer patients receiving targeted therapy in the training and validation cohorts.

Characteristic	Her2-positive Total (n=236), n (%)	Training cohort (n=158), n (%)	Validation cohort (n=78), n (%)	*P* value
**targeted therapy**				0.396
Single-agent targeted therapy	37 (15.68)	27 (17.09)	10 (12.82)	
Dual-agent targeted therapy	199 (84.32)	131 (82.91)	68 (87.18)	

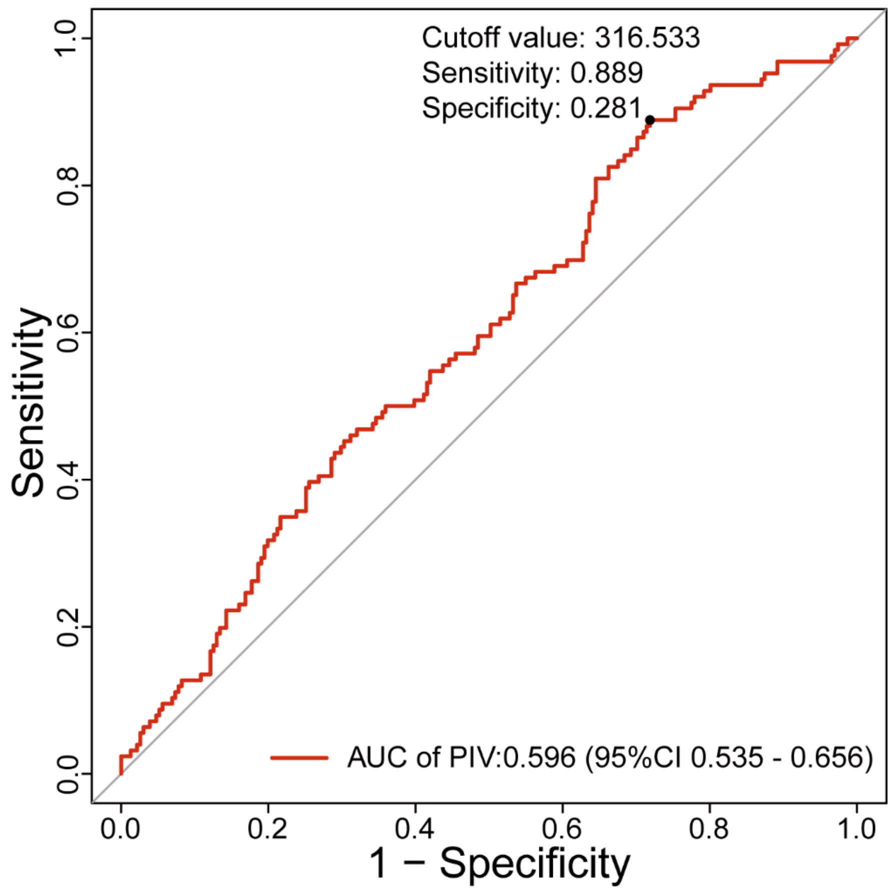

Figure S1. Identification of the best cut-off point of the pan-immune inflammation value (PIV)

Abbreviations: PIV: Pan-immune inflammation value; AUC: Area under curve; CI: Confidence interval

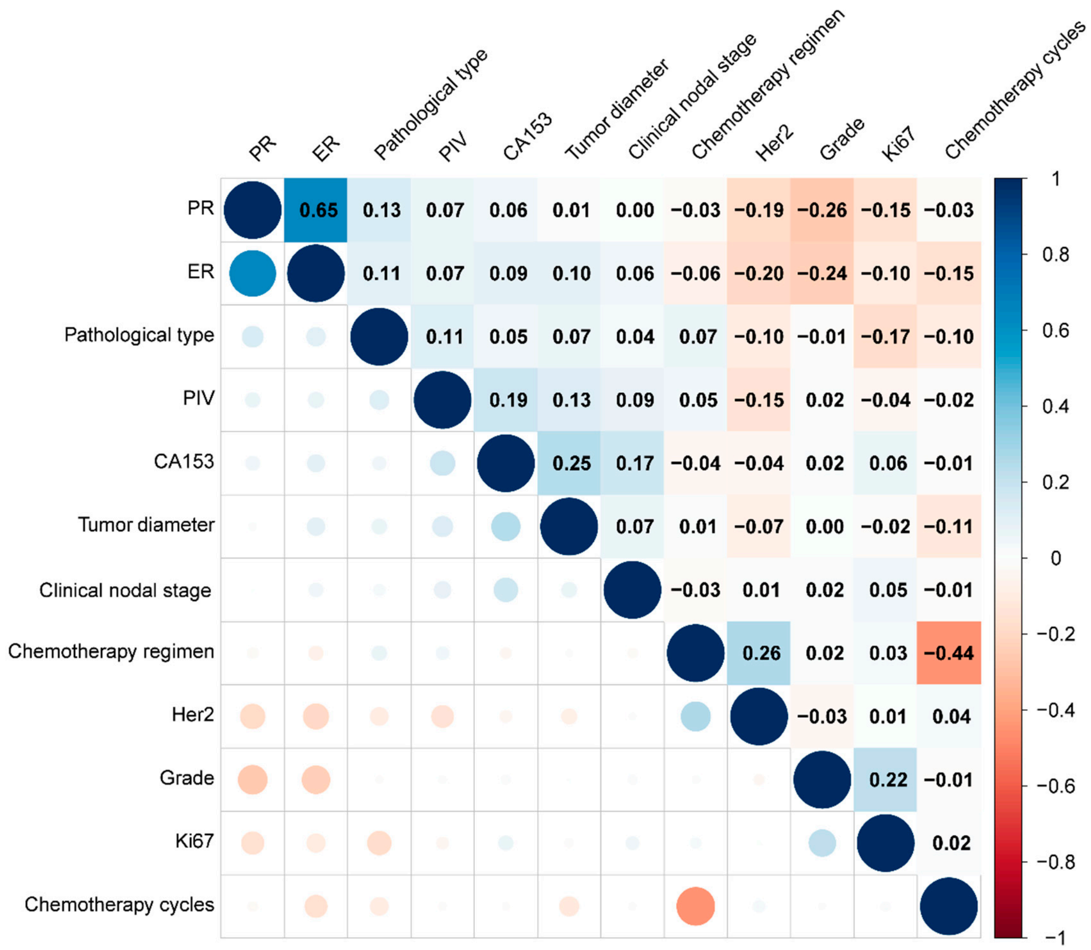

Figure S2. Constructing a correlation matrix of significant variables from univariate logistic regression to assess multicollinearity

Abbreviations: PIV: Pan-immune inflammation value; CA153: Carbohydrate antigen 15-3; ER: Estrogen receptor; PR: Progesterone receptor; Her2: Human epidermal growth factor receptor-2

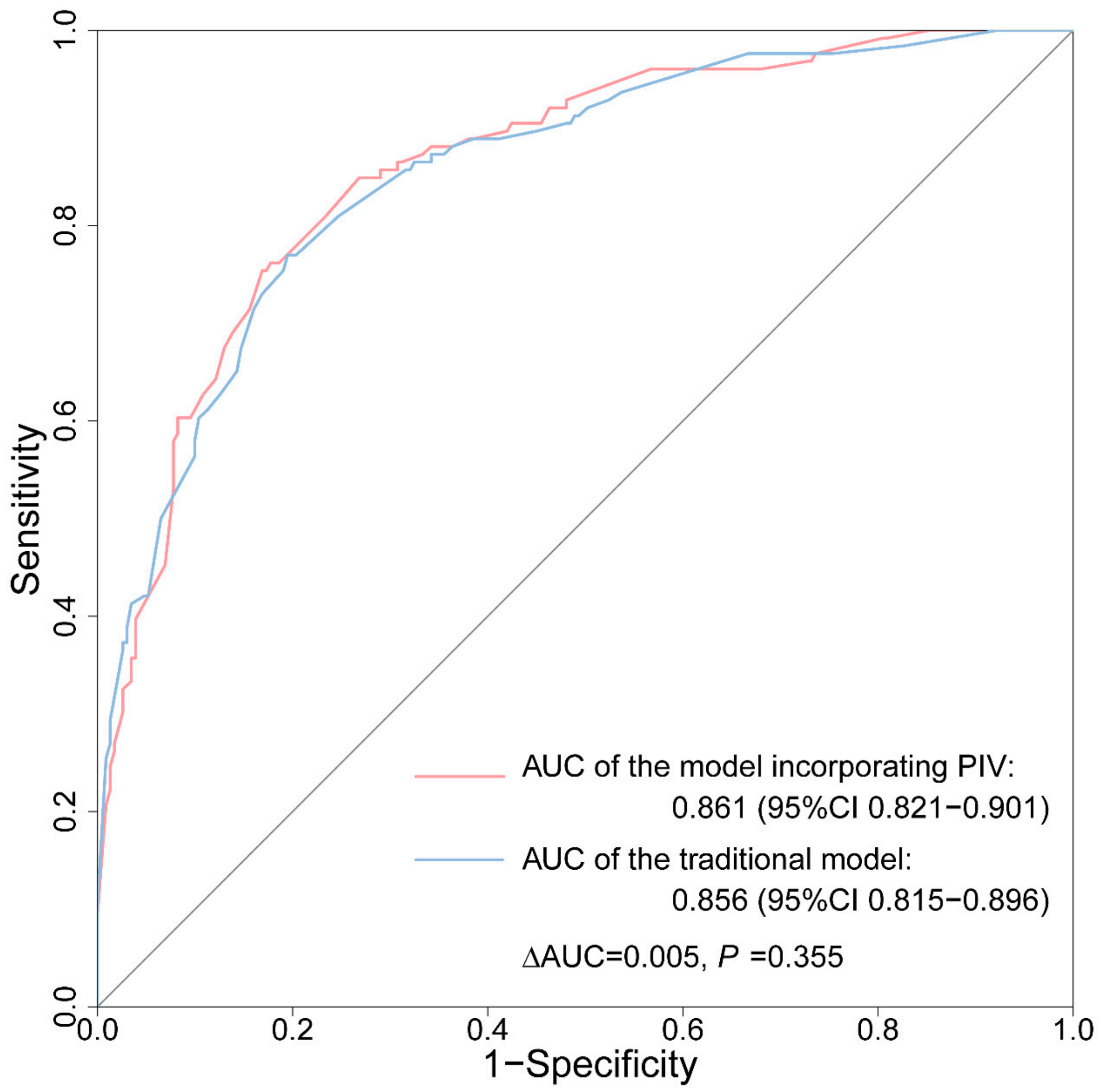

Figure S3. Receiver operating characteristic (ROC) curves of the combined model and the traditional model.

Abbreviations: PIV: Pan-immune inflammation value; AUC: Area under curve; CI: Confidence interval
